# Supplementary material for: High harmonic spectroscopy reveals anisotropy of the charge-density-wave phase transition in TiSe2
Source: Commun Mater. 2025 Jul 18;6(1):152. doi: 10.1038/s43246-025-00873-5 (PMC12270906; doi:10.1038/s43246-025-00873-5)
Supplement: Supplementary file 2 — Supplementary Information [file 43246_2025_873_MOESM2_ESM.pdf]

Supplementary: High harmonic spectroscopy reveals anisotropy of the Charge-Density-Wave phase transition in $TiSe_2$

Igor Tyulnev[1], Lin Zhang[1], Lenard Vamos[1], Julita Poborska[1], Utso Bhattacharya[2], Ravindra W. Chhajlany[3], Tobias Grass[4,5], Samuel Mañas-Valero[6], Eugenio Coronado[6], Maciej Lewenstein[1,7], Jens Biegert[1,7*]

[1]ICFO - Institut de Ciencies Fotoniques, The Barcelona Institute of Science and Technology, 08860 Castelldefels (Barcelona), Spain
[2]Institute for Theoretical Physics, ETH Zurich, 8093 Zurich, Switzerland
[3]ISQI - Institute of Spintronics and Quantum Information, Faculty of Physics and Astronomy, Adam Mickiewicz University, 61614 Poznań, Poland
[4]DIPC - Donostia International Physics Center, Paseo Manuel de Lardizábal 4, 20018 San Sebastián, Spain
[5]Ikerbasque - Basque Foundation for Science, Plaza Euskadi 5, 48009 Bilbao, Spain
[6]Instituto de Ciencia Molecular (ICMol), Universitat de València, 46980 Paterna, Spain
[7]ICREA, Pg. Lluís Companys 23, 08010 Barcelona, Spain
[*]Corresponding author : jens.biegert@icfo.eu

The reduced Brillouin zone scheme

A feature during charge density wave formation is the backfolding of the 1st Brillouin zone. It can be understood in real space as the formation of a supercell which in the case of $TiSe_2$ is commensurate with a 2x2x2 increase. In reciprocal space the equivalent description results in the connection between Γ and M points via the vectors $\mathbf{Q_1}$, $\mathbf{Q_2}$ and $\mathbf{Q_3}$ leading to the halving of the 1st Brillouin zone, see Supplementary Figure 1. Within the CDW phase the previous Γ and M points are thus equivalent and the new high-symmetry points (Γ, M, K) are marked with a bar. As the valence bands at Γ and the conduction bands at M now overlap renormalization occurs leading to the opening of the CDW gap between the valence band v_1 and c_1, c_2. The backfolded bands from the three $\mathbf{Q_i}$ directions renormalize differently, resulting in another higher energy transition from the valence band v_1 to conduction band c_3.

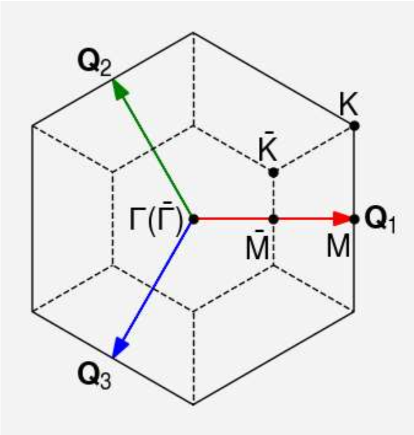

Supplementary Figure 1. Reciprocal space of $TiSe_2$. The dashed lines represent the reduced Brillouin zone folded by the CDW wave vectors Q_i. High symmetry points in the reduced and original Brillouin zone are denoted by symbols (Γ, M, K) with and without bar, respectively.

Optical conductivity

In order to obtain the optical conductivity for $TiSe_2$ in Fig. 1c, reflectivity curves adapted from [1] are fitted via a Drude-Lorentz oscillator model for the dielectric function of the form

$$\varepsilon(\omega) = \varepsilon_{\infty} - \frac{\omega_p^2}{\omega^2 + \mathrm{i}\,\omega/\tau} + \sum_{i=1}^{N} \frac{s_i^2}{\omega_i^2 - \omega^2 - \mathrm{i}\,\omega/\tau_i}$$

Where the number of Lorentz oscillators N consists of the phonon peaks in the low frequency regime and the transitions in the mid-infrared region. Focusing on the mid-infrared region, the fit parameters for the Drude term and two Lorentz terms are shown for completeness in Supplementary Table 1 at temperatures above and below the phase transition.

Temperature (K)	300	150	80	10
ω_p (cm^{-1})	7664±149	2450±58	2274±28	2351±22
τ_0 ($\mathrm{cm} \times 10^{-3}$)	$1.36\pm1.89\times10^{-8}$	3.93 $\pm3\times10^{-6}$	12.15 $\pm1.09\times10^{-5}$	17.9 $\pm2.9\times10^{-5}$
s_1 ($\mathrm{cm}^{-1} \times 10^3$)	25.89±69.9*	21.59±0.71	17.77±0.17	17.14±0.11
ω_1 (cm^{-1})	5497±1917*	3351±5.2	3421±0.42	3473±0.18
τ_1 ($\mathrm{cm} \times 10^{-3}$)	0.093 $\pm2.89\times10^{-9}$	0.17 $\pm1.1\times10^{-10}$	0.35 $\pm1.5\times10^{-10}$	0.42 $\pm1.46\times10^{-10}$
s_2 ($\mathrm{cm}^{-1} \times 10^3$)	51.49±10.5	53.72±0.58	55.49±0.5	56.24±0.56
ω_2 ($\mathrm{cm}^{-1} \times 10^3$)	13.93±0.025	13.82±0.013	13.6±0.014	13.61±0.016
τ_2 ($\mathrm{cm} \times 10^{-3}$)	$0.15\pm1.61\times10^{-10}$	$0.14\pm2.75\times10^{-11}$	$0.13\pm2.55\times10^{-11}$	$0.13\pm2.71\times10^{-11}$
ε_∞ (cm^{-1})	$18.47\pm3\times10^{-5}$	$18.53\pm2\times10^{-5}$	$18.53\pm2.5\times10^{-5}$	$18.55\pm2.9\times10^{-5}$

Supplementary Table 1. Fit parameters for the Drude-Lorentz model. Fits to the temperature dependent data adapted from Li et al. [1]. The Lorentz peaks consist of the oscillator strength s_i at the frequency ω_i and a damping rate τ_i related to the peak width. Additionally, ω_p is the plasma-frequency and ε_∞ the near constant value of the dielectric function in the high frequency limit. *Parameters for the first Lorentz oscillator have a high uncertainty at 300 K, as this peak only emerges below T_c.

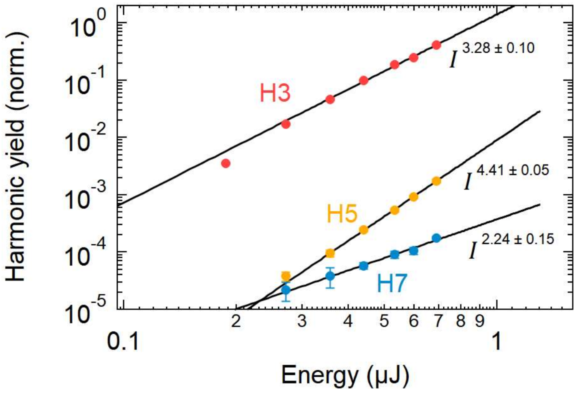

Supplementary Figure 2. Yield of harmonic orders 3, 5 and 7 as function of the driving field energy. Black lines are fits to extract the power-law for each harmonic. H3 and H5 agree well with the respective power laws in perturbation theory, while H7 has a significantly lower scaling.

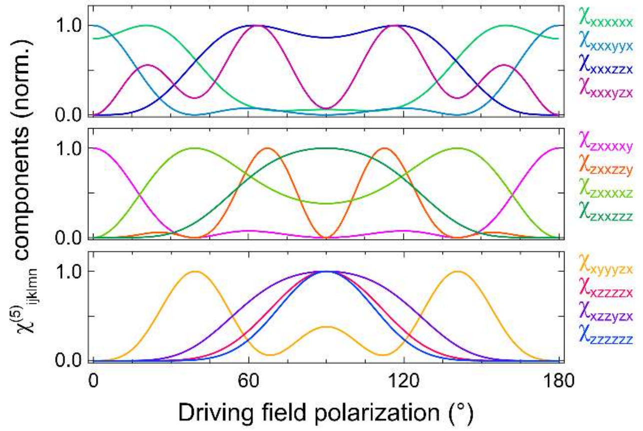

Supplementary Figure 3. Non-zero 5th order nonlinear susceptibility components as function of driving field polarization angle after projection for 45-degree incidence. All components are symmetric around 90 degrees. Out of 12 components only 2 produce a strict double peak feature at Γ-$M_{1,2}$ with 4 more influencing the contrast at 90 degrees.

Supplementary References

1. Li, G. *et al.* Semimetal-to-Semimetal Charge Density Wave Transition in 1T−TiSe2. *Phys Rev Lett* **99**, 027404 (2007).
